# Ionotropic Glutamate Receptors and Voltage-Gated Ca^**2+**^ Channels in Long-Term Potentiation of Spinal Dorsal Horn Synapses and Pain Hypersensitivity

**DOI:** 10.1155/2013/654257

**Published:** 2013-10-02

**Authors:** Dong-ho Youn, Gábor Gerber, William A. Sather

**Affiliations:** ^1^Department of Oral Physiology, School of Dentistry, Kyungpook National University, 188-1 Samduck-2, Chung-gu, Daegu 700-412, Republic of Korea; ^2^Department of Anatomy, Histology and Embryology, Semmelweis University, Tüzoltó Utca 58, Budapest, Hungary; ^3^Department of Pharmacology, University of Colorado School of Medicine, Mail Stop 8315, 12800 E. 19th Avenue, P18-7104, Aurora, CO 80045, USA

## Abstract

Over the last twenty years of research on cellular mechanisms of pain hypersensitivity, long-term potentiation (LTP) of synaptic transmission in the spinal cord dorsal horn (DH) has emerged as an important contributor to pain pathology. Mechanisms that underlie LTP of spinal DH neurons include changes in the numbers, activity, and properties of ionotropic glutamate receptors (AMPA and NMDA receptors) and of voltage-gated Ca^2+^ channels. Here, we review the roles and mechanisms of these channels in the induction and expression of spinal DH LTP, and we present this within the framework of the anatomical organization and synaptic circuitry of the spinal DH. Moreover, we compare synaptic plasticity in the spinal DH with classical LTP described for hippocampal synapses.

## 1. Introduction

 Long-term potentiation (LTP), an increase in the strength of synaptic transmission between neurons, has been proposed as a cellular model of learning and memory formation. Since LTP was first described for the dentate area of the hippocampal formation [1], data pertinent to mechanisms of LTP have been abundantly accumulated in diverse synapses of hippocampus and other brain areas. In contrast, investigation of LTP in the spinal dorsal horn (DH) [2] is more recent, beginning twenty years after the first description of LTP in the hippocampus, and spinal DH LTP has focused largely upon the synapses formed by primary sensory afferent fibers, because these synapses are the first checkpoint for pain signals entering the central nervous system (CNS). At these primary afferent synapses, LTP has been thought to be a cellular correlate of pain hypersensitivity and as such has been proposed as a potential target for therapeutic treatments of chronic pain.

Neurons in the spinal DH, consisting of superficial (laminae I and II) and deep (laminae III–VI) DH, receive synaptic inputs from primary afferent fibers, their cell bodies located within dorsal root ganglion (DRG) as well as those from other DH neurons, or neurons in other higher brain areas. The spinal DH neurons are considered as secondary neurons because peripheral somatosensory signals conveyed by primary sensory DRG neurons first reach these neurons. Synapses formed in these DH neurons mostly use glutamate for excitatory transmission. Generally, ionotropic glutamate receptors selectively activated by the artificial agonist *α*-amino-3-hydroxy-5-methyl-4-isoxazolepropionate (AMPA) support the largest component of glutamatergic excitatory synaptic transmission in the CNS, while the *N*-methyl-D-aspartate (NMDA) receptor subtype is most important in the induction of synaptic plasticity, including LTP (*see below*). In addition to ligand-gated excitatory ion channels, DH neurons express various types of voltage-gated ion channels that generally contribute to neuronal excitability. Among the voltage-gated ion channels, voltage-gated Ca^2+^ channels (VGCCs) have been found to be involved in the control of synaptic plasticity, owing to their control of Ca^2+^ influx into both presynaptic nerve terminals and postsynaptic domains of neurons. 

In this paper, we review the contributions of these two classes of ion channels to LTP in the spinal DH area. To provide a context for interpretation of the role of these channels in LTP, we first briefly discuss the anatomical organization and synaptic circuitry of the spinal DH and also consider synaptic transmission and plasticity in the spinal DH. For the sake of brevity, this review does not consider the roles of other types of ion channels in plasticity and pain, nor does it focus upon downstream signaling pathways known to be critical for LTP.

## 2. The Spinal Cord Dorsal Horn

### 2.1. Anatomical Organization

The DH of the spinal cord can be subdivided into six distinct layers (laminae I–VI) in the dorsal-ventral direction of the gray matter, which was first proposed in cat [3] as well as in rat [4]. The Rexed laminae I and II consist of superficial spinal DH [5], and laminae III–VI are frequently called deeper layer of the spinal cord. Due to concentrated small neurons and their processes plus a relative small number of myelinated axons, the lamina II is observed as a translucent band under the naked eye or light microscope and is called “substantia gelatinosa (SG)” [4, 6]. Lamina VI exists only in the cervical and lumbosacral enlargements [3]. Generally, the spinal DH consists of the central terminals of primary sensory neurons, projection neurons, intrinsic DH neurons, and descending nerve fibers from the brainstem and other higher brain structures. The cell bodies of the primary sensory neurons are located in the DRG. Each ganglion cell sends an axon that branches into a peripheral process and a central process. The peripheral process contributes to a peripheral nerve and terminates peripherally as a sensory receptor. The central process enters into the spinal cord through a dorsal root and further branches to numerous collaterals. Together, these two processes form primary afferent fibers that transmit encoded information from periphery to the spinal cord or trigeminal nuclei of the brain stem.

Although primary afferent fibers give off most of their collaterals to the segment of the spinal cord that they enter, they also spread in the rostrocaudal direction. The distribution of primary afferent fibers in the spinal DH is in an orderly way based on fiber size, which affects conduction velocity and sensory modality [7]. Most fine myelinated (A*δ*; conduction velocity, 1–1.5 to 5–10 m/sec) or unmyelinated (C; <1–1.5 m/sec) primary afferent fibers end predominantly in laminae I and II, although a few reach laminae III–VI [8, 9]. In detail, high threshold A*δ* mechanoreceptors terminate in laminae I and V, while low threshold A*δ* mechanoreceptors only terminate in lamina III [9]. Most large cutaneous afferents (A*β*; >5–10 m/sec), which function as low threshold mechanoreceptors, have a characteristic pattern of termination in the deeper laminae (III–VI) of the DH [10]. Cutaneous C fibers, occupying ~80% of cutaneous primary afferent fibers [11] and the majority of which being high-threshold polymodal nociceptors in the rat [12], terminate in lamina II [13–15], although there is also a contribution to lamina I [16]. Based on neurochemical markers, the high-threshold C fibers can be divided into two major groups: peptidergic and nonpeptidergic [7]. Peptidergic C fibers are nociceptors [17] and contain neuropeptides such as calcitonin gene-related peptide (CGRP) and/or substance P and express TrkA or transient receptor potential (TRP) V1 [18]. The peptidergic substance P-containing C fibers ends mainly in lamina I and the outer layer of lamina II (IIo). It is estimated in lumbar DRG of rat that approximately half of the C fibers are peptidergic [19]. Other high-threshold C fibers do not contain peptides, but most of them can be revealed by their ability to bind the lectin Bandeiraea simplicifolia isolectin B4 (IB4) [20], and a subpopulation of the nonpeptidergic C fibers can be defined by Mas-related G-protein-coupled receptor member D (MrgprD), a sensory neuron-specific G-protein-coupled receptor [21]. Although the function of nonpeptidergic C fibers is poorly understood, this population also includes many nociceptors [22, 23] and is different from the peptidergic C fiber because ablation of the MrgprD afferents in adult mice results in a selective loss of sensitivity to noxious mechanical (but not thermal) stimuli [7, 24].

Beside the high-threshold C fibers, it should be pointed out that there are low-threshold mechanosensitive C fibers, which are nonpeptidergic and innervate specific types of hair follicles [25]. Interestingly, this type of nonpeptidergic C fiber expresses neither IB4 nor MrgprD but exclusively expresses tyrosine hydroxylase, the enzyme catalyzing L-3,4-dihydroxyphenylalanine production and participates in forming narrow unique columns in the spinal DH with other low-threshold A*δ* and A*β* fibers [25].

### 2.2. Synaptic Circuitry

**Figure 1:**
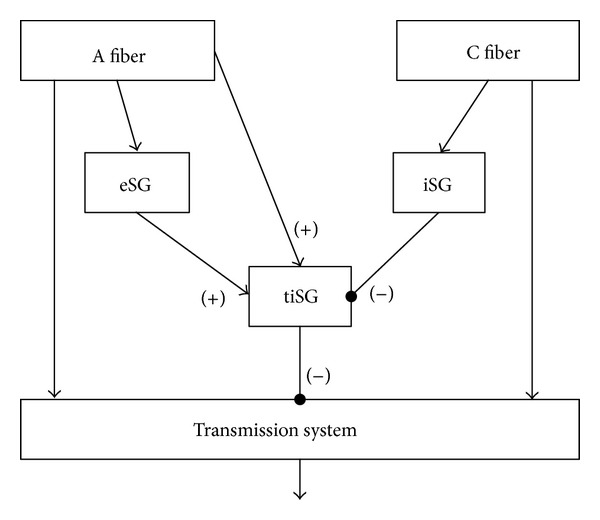
A diagram modified from the gate control theory. Both primary afferent A and C fibers directly target the transmission system that conveys the pain signals from the spinal dorsal horn to the higher brain areas. However, both fibers differentially innervate to the substantia gelatinosa (SG) neurons in the spinal DH. Although polysynaptic inputs are possible in all SG neurons from primary afferent fibers and other SG neurons, monosynaptic inputs from A fibers reach the excitatory SG neurons (eSG) and the transmission-inhibiting SG neurons (tiSG), while those from C fibers only go into the inhibitory SG neurons (iSG), not the itSG directly. The tiSG neurons receive the excitatory synaptic inputs from the eSG and the inhibitory synaptic inputs from the iSG. The main function of tiSG is inhibiting the transmission system, both presynaptically (in the gate control theory) and postsynaptically (in this diagram). In this way, the activation and inhibition of SG neurons (here, called tiSG) by large-diameter and small-diameter fibers, respectively, are possible, shown in the gate control theory.

The gate control theory, proposed by Melzack and Wall [26], illustrates how pain signals are transmitted to higher brain areas via the spinal DH. In this theory, inhibitory SG neurons control presynaptically both large- and small-diameter fibers, presumably corresponding to, respectively, A and C fibers, and these in turn innervate the transmission system. Therefore, activation of SG neurons by large-diameter fibers attenuates signals conveyed via both fiber types to transmission system neurons, which corresponds to gate closing; in contrast, inhibition of SG neurons by small diameter fibers opens the gate for transmission of pain information. Although the theory proposes a prominent role for SG neurons in gating pain transmission, it assumes only a single type of SG neurons, which is certainly incorrect in regard to the synaptic organization of the DH [27]; rather, recent data demonstrate that different subtypes of SG neurons are present in spinal DH and that these subtypes make distinct contributions to the function of the complex synaptic network of the spinal DH. Thus, based on recent morphological and electrophysiological studies in the spinal DH, we attempt to assign various SG neurons to three functionally different SG neurons: inhibitory SG (iSG) neurons, excitatory SG (eSG) neurons, and transmission system-inhibiting (tiSG) SG neurons (Figure 1). Large-diameter A fibers directly, or indirectly through eSGs, activate tiSG neurons, whereas small-diameter C fibers necessarily activate iSG neurons to inhibit the tiSG neurons. The transmission system is composed of projection neurons that send pain information to higher brain centers. In future works, it will be important to more completely describe the synaptic organization of the DH, and it will be important to carefully define the three basic classes of SG neurons.

The primary candidates for iSG neurons are *γ*-aminobutyric acid- (GABA-) ergic neurons in the spinal DH, an electrophysiologically heterogeneous group [28, 29] that make up approximately 30% of neurons in lamina II (SG) of the spinal cord [30]. It has been suggested, using combined single/paired whole-cell patch-clamp recordings and biocytin labeling, that ~75% of all SG neurons fall into five groups that differ in firing behavior and other electrophysiological properties and most prominently, in the structure of their dendritic arbors: islet, radial, central, medial-lateral, and vertical cells [31–36]. Among these groups, islet cells are exclusively GABAergic [37, 38] and receive monosynaptic input from C fibers and polysynaptic input from A*δ* fibers [33, 35, 36]. The GABAergic nature of islet cells corresponds with the finding that, among SG neurons expressing GFP (driven by the upstream regulatory sequence of the gene encoding the GABA synthetic enzyme, glutamic acid decarboxylase (GAD) 67), 62% have islet-type morphology [29]. Because the islet cells do not directly target projection neurons [34] yet do influence the pain transmission system through other SG neuronal types, islet cells are apparently one of the major sources of SG neurons that act as iSG neurons (Figure 1).

In addition to the islet-type of SG neuron, tonically firing neurons with central-type dendritic arbors are also likely GABAergic [32]. Central neurons receiving GABAergic input from islet cells and glutamatergic input from C fibers are divided into either tonically firing neurons or transiently firing neurons [31] and are both excitatory and inhibitory [32–35]. The tonically firing central neurons synapse on vertical neurons and recurrently, on islet cells, identifying this subtype of central neuron as an additional type of iSG neuron.

As noted above, 30% of SG neurons are GABAergic so that ~2/3 of SG neurons are glutamatergic; eSG neurons are drawn from this large pool of excitatory interneurons. The eSG neurons likely include vertical and radial SG neurons because they predominantly receive monosynaptic inputs from A fibers [34, 36, 38]. In contrast, it is known that central-type SG neurons do not receive monosynaptic inputs from A fibers, thus ruling out central neurons as candidate eSG neurons.

On the other hand, considerably less is known about the types of GABAergic SG neurons that make up the tiSG circuit component, which synapse on projection neurons in lamina I and deeper laminae [7]. Neurokinin (NK) 1-positive projection neurons in laminae III-IV are known to receive inputs from GABAergic neurons that contain neuropeptide Y (NPY) [39]. Although classification of SG neuronal types by arborization pattern and electrophysiological properties remains incomplete, because NPY-expressing neurons comprise half of the GABAergic neurons of laminae I and II [40], we suggest that these NPY-positive neurons inhibit the transmission system and thus may act as tiSG neurons (Figure 1). Therefore, careful identification of the types of (1) primary afferent fibers and (2) local interneurons that send inputs to NPY-positive, GABAergic neurons, would greatly clarify the synaptic circuitry that contributes to gating of pain signals. Evidence for another type of tiSG neuron has also been obtained: “giant marginal” projection neurons, which lack NK1 receptors and express the glycine receptor-associated protein gephyrin [41], are richly contacted by GABAergic boutons that contain nitric oxide synthase (NOS) but not NPY [41]. Here again, the neuronal morphology needs to be defined for this NOS-positive neuron. Altogether, clear understanding of the synaptic circuitry underlying gating of pain signals will require a more complete description of the pattern of SG neuron connectivity with GABAergic, NPY- or NOS-positive neurons.

### 2.3. Synaptic Transmission

At chemical synapses in the CNS, neurotransmitters released from presynaptic nerve terminals generate graded analogue signals through the opening of ligand-gated ion channels on the plasma membrane of postsynaptic neurons. Whereas each presynaptic neuron possesses the biochemical machinery to release only one type of neurotransmitter, which can be either excitatory or inhibitory, individual postsynaptic neurons express a variety of ligand-gated ion channels that respond to different neurotransmitters so that postsynaptic neurons can, for example, exhibit both excitatory and inhibitory synaptic potentials. In the CNS, glutamate generates fast excitatory synaptic signals in postsynaptic neurons by opening any of three types of ligand-gated glutamate receptor ion channels: based on their pharmacology and structural homology, these are known as AMPA receptors (subunit: GluA1-GluA4), kainate (subunit: GluK1-GluK5) receptors, and NMDA receptors (subunit: GluN1, GluN2A-GluN2D, GluN3A, and GluRN3B) [42].

It is known from early studies of the spinal DH that glutamate-mediated fast excitatory synaptic transmission involves activation of postsynaptic ionotropic glutamate receptors [43–46]. AMPA receptors mediate the large early component of fast excitatory synaptic postsynaptic responses, whereas the more slowly opened NMDA receptors contribute only to the later component of excitatory postsynaptic responses [2, 45, 46]. Fast synaptic transmission mediated by kainate receptors is relatively small and produces slowly decaying synaptic currents [47, 48]. Although most spinal DH synapses use all three classes of ionotropic glutamate receptors, some synapses possess only NMDA receptors; these are known as “silent synapses” because, lacking AMPA-receptor-driven postsynaptic depolarization, the glutamate-activated NMDA receptors at these synapses remain blocked by Mg^2+^ and thus fail to generate a synaptic signal [49, 50].

Although inhibitory transmission falls outside the scope of the present review, we note that many interneurons in the spinal DH release GABA and glycine, which provide fast inhibitory transmission that is an essential feature of spinal DH circuits. Most GABAergic neurons also release glycine in the spinal DH [30, 51, 52], but GABA-mediated transmission can be distinguished from glycine-mediated transmission based on the decay kinetics of synaptic responses [53, 54].

### 2.4. Synaptic Plasticity

Strength of synaptic transmission in the CNS is not constant; rather, it is subject to up- or downmodulation as a consequence of patterns of presynaptic and/or postsynaptic activity. Such activity-dependent changes in synaptic strength are accomplished, in part, through long-term modulation of the properties and numbers of ion channels that mediate, affect, or respond to synaptic activity. LTP—a persistent increase in the strength of synaptic transmission [1]—can be induced by tetanic stimulation, pairing of presynaptic activity with postsynaptic depolarization, coincidence between presynaptic release of glutamate and postsynaptic depolarization, and pharmacological treatments that increase excitatory postsynaptic responsivity. Since the initial discovery of LTP, molecular and cellular mechanisms subserving this kind of plasticity have been worked out most clearly for the canonical form of NMDA receptor-dependent LTP that is found at Schaffer collateral-commissural synapses onto pyramidal neurons in the CA1 region of hippocampus. Important elements that have been identified include channel phosphorylation by protein kinases such as protein kinase A (PKA), protein kinase C (PKC), and Ca^2+^-calmodulin-dependent protein kinase II (CaMKII) [55–59]; consequent increases in channel opening probability and single-channel conductance [60, 61]; subunit-specific trafficking of postsynaptic AMPA receptors [62–64] to the subsynaptic membrane; and changes in glutamate release, both in probability [65] and quantal content [66] at presynaptic terminals [67]. The cellular processes most carefully worked out for hippocampal LTP [68] are generally thought to provide a fundamental basis for information processing and storage throughout CNS and particularly for learning and memory in the hippocampus [69–72].

In the spinal cord DH, early studies revealed that repetitive stimulation of dorsal root or peripheral nerve produces LTP at primary afferent synapses [2, 73, 74]. In addition to involvement of NMDA receptors and postsynaptic Ca^2+^ that is typical of LTP induction in hippocampus [2, 75], spinal DH studies have identified roles for NK1 [75, 76], group I metabotropic glutamate [77], and opioid [78] receptors in the induction and expression of LTP [2].

Some patterns of synaptic activity can cause a decrease in synaptic strength, referred to as “long-term depression (LTD)” [79]. This type of synaptic plasticity has also been extensively studied in various CNS regions, most particularly in the context of certain forms of information processing in the hippocampus [80, 81] and also of motor learning in cerebellum [82]. Although both high-frequency stimulation (HFS) and low-frequency stimulation (LFS) can induce LTD in the spinal DH, protein phosphatases play a role only in the induction of HFS-induced LTD [83] but not in that of LFS-LTD [84] in this area.

Because spinal LTP and LTD may play critical roles in hyperalgesia and allodynia [85, 86] and the activation of high-threshold C fibers is important to mediate many type of hyperalgesia, C fiber-mediated field potentials have been the subject of many LTP studies. LTP of C fiber-evoked field potentials is reliably produced by HFS of peripheral nerves (>3 hours), and is dependent upon the activation of NMDA receptors [75]; interestingly, LFS at C fiber intensity also induces LTP under certain conditions [87, 88]. Moreover, C fiber-mediated LTP can be induced by noxious stimulation or injury [89], revealing a contribution of this form of synaptic plasticity to induction of hyperalgesia. Although the loci of mechanisms underlying the LTP of C fiber-evoked field potential are difficult to clarify, the induction and/or maintenance of this type of LTP involve many channels and signaling molecules, including NMDA and NK1 receptors [75], N-type and P/Q-type VGCCs [90], TRPV1 channels [91], the EphB receptor tyrosine kinase [92], ryanodine receptors [93, 94], nitric oxide [95, 96], and many inflammatory agents [97].

Spinal LTP has also been studied using single (whole) cell recordings of primary afferent stimulation-evoked excitatory postsynaptic potentials or currents (EPSPs and EPSCs, resp.). In this case, A*δ* fiber- and C fiber-mediated synaptic responses can be distinguished according to stimulus intensity and conduction velocity, which is advantageous in elucidating primary afferent-dependent mechanisms [87, 98, 99] or the locus of induction of LTP [93, 100]. In addition, whole cell recordings are advantageous for study of LTP in particular types of DH neurons. Combining whole cell recording from DH neurons with injection of retrograde tracers (e.g., DiI) into the parabrachial (PB) area or periaqueductal gray (PAG) has allowed researchers to determine that HFS induces LTP of C fiber-mediated EPSCs in lamina I neurons that project to PB area but not in those that project to PAG [87]; in contrast, LFS induces LTP in lamina I neurons that project to PAG area. LTP of C fiber-EPSCs by HFS in the PB-projecting lamina I neurons requires NK1 receptor-mediated signaling and activation of T-type VGCCs [99]. LFS-induced LTP of C fiber EPSCs in the PAG-projecting lamina I neurons requires nitric oxide signals [87, 93], the latter generated in response to intracellular Ca^2+^ rises that are slower in onset and prolonged in comparison to the kinetics of Ca^2+^ rises required to elicit LTP in PB-projecting lamina I neurons [87].

In CA1 of hippocampus, LTP of EPSCs can be alternatively induced by the coordinated activity of presynaptic fibers and postsynaptic neurons, which is characteristic of spike-timing [101] or pairing protocols [102] in the hippocampus. The spike-timing protocol requires low-frequency postsynaptic spikes timed within 10 ms of the onset of synaptic responses, while the pairing protocol involves persistent postsynaptic depolarization (0~+30 mV) during repetitive low-frequency presynaptic stimulation. These protocols have been applied to study LTP of EPSCs in the spinal DH as well, in an effort to overcome the low rate of success (~50%) observed for the induction of LTP by HFS [2]. Spike-timing dependent LTP is interestingly dependent on expression of the GluK2 (previously known as GluR6) kainate receptor subunit [48], along with activation of NMDA receptors and elevation of intracellular Ca^2+^ [103]. In spinal DH, LTP of EPSCs by the pairing protocol requires activation of extracellular signal-regulated kinase (ERK) [104].

Considering the mixture of excitatory and inhibitory interneurons and the complex synaptic circuitry in lamina II of the spinal DH (see above), the contribution of LTP to hyperalgesia will necessarily depend upon which synapses in the DH circuitry specifically undergo LTP. It is therefore critical that LTP and its pathophysiological role in pain hypersensitivity be pursued at identified types of SG neuron synapses. For example, hyperalgesia may be produced by LTP of excitatory interneurons that synapse upon neurons that, in turn, project to brain areas involved in nociception. Alternatively, hyperalgesia may be reduced by LTP of synapses onto inhibitory interneurons that target projection neurons. Hence, careful identification of the specific subtype of neuron studied will be essential to better understand the roles of spinal DH LTP in hyperalgesia and allodynia [85].

## 3. Contribution of Ionotropic Glutamate Receptors to LTP in the Spinal DH

### 3.1. AMPA Receptors

AMPA receptors consist of homo- and heterotetrameric assemblies of GluA1, 2, 3, and 4 subunits, with different assemblies of AMPA receptor subunits exhibiting distinct functional behaviors [42, 105]. Among the GluA subunits, transcripts encoding GluA2 are subject to RNA editing at position 586, which results in replacement of the neutral glutamine (Q) residue found in all other GluA subunits with a positively charged arginine (R). Position 586 is located in transmembrane segment 2 (M2), which forms the lining of the ion permeation pathway through the receptor; an arginine at this position decreases the receptor's Ca^2+^ permeability and also confers linear current-voltage behavior. AMPA receptors lacking GluA2 subunits are significantly more Ca^2+^-permeable, exhibiting a Ca^2+^ permeability ratio (*P*_Ca_/*P*_Na_) of 3, and they also display strong inward rectification in their current-voltage relationships [105, 106].

Because AMPA receptors are the main mediators of excitatory synaptic transmission in the CNS, they are generally considered as the final target for induction and expression of LTP, rather than as inducers or regulators. Thus, principal endpoints in LTP are phosphorylation and trafficking of specific AMPA receptor subunit subtypes, along with changes in AMPA receptor conductance [42, 105]. The GluA1 subunit, for example, can undergo phosphorylation of Ser831 by CaMKII [107] and PKC [108] and of Ser845 by PKA [108], which contributes to induction of LTP by changing the open probability and single-channel conductance of AMPA receptors containing this subunit [42]. In regard to membrane trafficking of AMPA receptors, the induction mechanism for LTP in hippocampal CA1 pyramidal neurons [64] includes increased incorporation of GluA1/GluA2-containing AMPA receptors into the synaptic surface membrane [62, 109]; however, subsequent work suggests that the newly incorporated AMPA receptors are in fact homotetrameric GluA1 complexes [110]. In accordance with these studies, the induction of LTP at hippocampal CA1 synapses is impaired in mice deficient in the GluA1 subunit [111]. Surface membrane incorporation of homomeric GluA1 receptors may result in the replacement of preexisting GluA2-containing AMPA receptors, thereby increasing the net Ca^2+^-permeability of the AMPA receptor population in the postsynaptic surface membrane [112]. Increased Ca^2+^ influx via GluA2-lacking, Ca^2+^-permeable AMPA receptors, is directly related to enhancement of LTP [113, 114].

Trafficking of AMPA receptors requires their interaction with transmembrane AMPA receptor regulatory proteins (TARPs) [115, 116]. Interaction of TARPs with AMPA receptors prevents AMPA receptor degradation [117], and subsequent interaction of AMPA receptors with PSD-95 results in translocation of AMPA receptors from the perisynaptic region into synaptic sites [118]. In contrast to these studies, a recent study has found that the GluA1 C-terminal tail, critical for GluA1 trafficking [109, 110], is not required for LTP [119]. This has led to the suggestion that a reserve pool of AMPA receptors, regardless of their subunit composition, is relied upon for LTP. Further studies are needed to provide a more comprehensive picture of the mechanism and role of AMPA receptor trafficking in hippocampal LTP. In addition, studies of this process in spinal DH LTP remain to be carried out.

Ca^2+^-permeable AMPA receptors are expressed in inhibitory interneurons [120] of lamina I and the outer layer of lamina II [121], the laminae which receive synaptic input primarily from nociceptive C fiber afferents. Ca^2+^-permeable AMPA receptors in layer I and II DH neurons are activated by synaptic input [122], raising the possibility that these channels play a special role in mediating sensory input by unmyelinated fibers [123]. Using GluA2 knockout mice, it has been shown that Ca^2+^-permeable AMPA receptors enhance HFS-evoked LTP and mediate induction of NMDA receptor-independent LTP at primary afferent-DH neuron synapses [98], which suggests that Ca^2+^-permeable AMPA receptors contribute significantly to LTP in the spinal DH and may substitute for NMDA receptors in LTP induction. In contrast to NMDA receptors, Ca^2+^-permeable AMPA receptors allow Ca^2+^ influx at resting membrane potential, a potential merit for induction of synaptic plasticity.

### 3.2. NMDA Receptors

Functional NMDA receptors are heterotetrameric assemblies composed of two GluN1 subunits and either two GluN2 subunits or a combination of GluN2 and GluN3 subunits [42]. The glutamate binding sites are located in the GluN2 subunits [124] and the glycine binding sites in the GluN1 and GluN3 [124–126]. NMDA receptors are characterized by their high permeability to Ca^2+^ [127], voltage-dependent block by Mg^2+^ [128], and slow “activation/deactivation” kinetics [129]. NMDA receptor alternative splice variants exhibit subtle differences in functional properties, thereby fine-tuning the behavior of NMDA receptors in which they are incorporated [130]. For example, NMDA receptors containing GluN2A or GluN2B subunits display high-conductance channel openings and a high sensitivity to block by extracellular Mg^2+^, whereas receptors composed of GluN2C or GluN2D subunits show low-conductance openings and lower sensitivity to Mg^2+^. Moreover, GluN1/GluN2A-containing NMDA receptor currents deactivate rapidly (time constant of tens of milliseconds), whereas GluN1/GluN2D-containing NMDA receptor currents deactivate very slowly (time constant of several seconds) [131–133]. In addition, GluN3 can also coassemble with GluN1 [134–136] to form uniquely excitatory glycine receptors [136]. These distinctive properties may provide particular NMDA receptor subtypes with specific roles in excitatory synaptic transmission/plasticity and pathology.

 The role of NMDA receptors in the induction of LTP is well established for various brain synapses, particularly the Schaffer collateral input to CA1 pyramidal neurons in hippocampus [68–70]. The activation requirements for NMDA receptors—agonist (glutamate) binding and postsynaptic depolarization—are well-matched to the “Hebbian” properties of LTP induction, namely, specificity, associativity, and cooperativity [69]. Further, several recent studies show that a proper subunit composition is essential for the induction [137–139] and expression [140, 141] of LTP. These results reflect the fact that specific NMDA receptor subunits are differentially phosphorylated by various protein kinases, such as src [142, 143] and also differentially interact with other accessory and regulatory proteins [144]. In keeping with the notion of a proper NMDA subunit composition in LTP, the association of active CaMKII with GluN2B is likely required for the induction of canonical LTP at Schaffer collateral synapses on CA1 neurons [145]. Downstream of these regulatory processes, NMDA receptor-dependent protein synthesis [146–148] is needed for the expression of LTP that persists beyond ~4 hours, referred to as late-LTP [149].

In the spinal DH,* in situ* hybridization or immunostaining has revealed high expression of GluN1 and GluN2D [150, 151] and lower levels of GluN2A and GluN2B [152, 153]. Electrophysiological measurements of conductance ratio have shown that lamina II GABAergic interneurons express both the GluN2A/GluN2B- and GluN2C/GluN2D-containing NMDA receptors, while excitatory lamina II interneurons express primarily GluN2A/GluN2B-containing receptors [154]. In addition, outside-out patch recordings of single channel currents have shown that, at least in extrasynaptic regions, both GluN1/GluN2B (high conductance; 57 pS) and GluN1/GluN2D (low conductance; 44 pS and 19 pS) are present on spinal DH neurons [155].

In the spinal DH, induction of nearly all forms of LTP is dependent on the NMDA receptors [86]. An early report showed that HFS (100 Hz) of primary afferent fibers at C fiber-activating intensity induces LTP of EPSPs in transverse spinal cord slices in vitro; LTP was absent in the presence of the NMDA receptor antagonist D-2-amino-5-phosphonovalerate (D-AP5) [2]. In addition, LTP induction at C fiber synapses also requires activation of NMDA receptors [73, 87, 99]. Recently, LFS (2 Hz at C-fiber intensity) of sciatic nerve has been shown to induce LTP of C fiber-evoked field potentials. This LFS-induced LTP is also prevented by an NMDA receptor antagonist, MK-801 in these experiments [156]. As expected, the noble anesthetic gas xenon, which has an inhibitory effect on NMDA receptors [157], prevents induction of LTP at C fiber synapses in intact rats [158]. LTP can also be induced by chemical means, for example, by perfusion of spinal cord slices with NMDA (+ postsynaptic depolarization) [159] or by perfusion of spinal cord segments with NMDA in spinalized, deeply anesthetized adult rat [75]. Taking together, these findings indicate that the NMDA receptor is required for induction of LTP in synapses of primary afferent fibers onto spinal DH neurons.

### 3.3. Kainate Receptors

Kainate receptors are tetramers assembled from combinations of five different types of subunits, termed GluK1-5 (formerly, GluR5-7 and KA1-2) [42, 105, 106, 160]. Each kainate receptor monomer possesses a ligand-binding site and a distinctive amino acid sequence that forms the channel lumen. Radioligand binding assays indicate that GluK1, 2, and 3 contribute to low-affinity kainate binding sites (K_D_ of 50–100 nM) [161], whereas GluK4 and 5 form high-affinity kainate binding sites (K_D_ of ~4–15 nM) [162, 163]. Structural variability of kainate receptors is conferred by alternative splicing and RNA editing [160]. Alternative splice variants have been found exclusively for GluK1 (GluK1-1, 1-2a, 1-2b, and 1-2c) [164] and GluK3 (GluR3a and 3b) subunits [165] in rat; however, the mouse GluK2 exists as two splice variants that differ in their C-terminal domains [166]. RNA editing, as for GluA2 subunits, posttranscriptionally modifies a Q/R site in the M2 segment of GluK1 and GluK2 subunits. The Q-to-R substitution in GluK2 homomeric kainate receptors decreases Ca^2+^ permeability [167, 168] and increases Cl^−^ permeability [169], reduces unitary conductance, and transforms channels from inwardly rectifying to linear or slightly outwardly rectifying. Mice deficient in Q/R editing in GluK1 have been found to exhibit a reduction in kainate receptor-mediated currents in DRG neurons [170], although the responses of these animals to painful stimuli are unaffected. Besides the Q/R editing site, two additional positions prone to RNA editing have been identified in the GluK1 subunit: an isoleucine (I)/valine (V) site and a tyrosine (Y)/cysteine (C) site [171], both in the M1 segment. Although the additional editing sites may modulate Q/R site control of Ca^2+^ permeability, the mechanism of interaction among the three editing sites remains to be elucidated.

For NMDA receptor-independent LTP at the mossy fiber-CA3 synapse in hippocampus, there is disagreement regarding the role of kainate receptors [172]. A selective antagonist for GluK1-containing kainate receptors, LY382884, blocks the induction of mossy fiber LTP [173, 174], but conflictingly, mossy fiber LTP can be elicited in the presence of the AMPA/kainate receptor antagonist, CNQX [175, 176]. Knockout mice deficient in GluK2 subunits [177] display reduced mossy fiber LTP, but mice deficient in GluK1 possess normal LTP. Although much more work is needed, the results point to a potential role for kainate receptors in mossy fiber LTP, specifically in the NMDA receptor-independent form of LTP.

For synapses of primary afferents onto spinal DH neurons, fast EPSCs have been shown to be mediated by postsynaptic kainate receptors [47]. Kainate receptor-mediated EPSCs are much smaller in peak amplitude and slower in decay kinetics than those mediated by AMPA receptors. To date, the kainate receptor subunits mediating synaptic transmission have not been well-characterized. However, low expression of GluK1 subunits, moderate expression of GluK3 and GluK4, and strong expression of GluK5 have been found for spinal DH neurons; no expression of GluK2 has been detected [150, 178]. Despite the apparent absence of GluK2 subunits from DH neurons, kainate receptor-mediated whole cell current and synaptic potentials recorded from spinal DH neurons are significantly decreased in GluK2 mutant mice [48]. In addition to expression on postsynaptic membrane in spinal DH neurons, kainate receptors are also expressed on DRG neurons, including primary afferent presynaptic terminals within the DH [179, 180]. All kainate receptor subtypes are present in DRG neurons, with GluK1 expressed at an especially high level [178, 181–183]. GluK1- or GluK2-containing presynaptic kainate receptors modulates glutamatergic transmission at A*δ* and C-fiber primary afferent-activated synapses in the spinal SG [184]. Interestingly, induction of LTP is impaired in GluK2 knockout mice, while the late phase of LTP is impaired in GluK1 mutant mice [48], indicating differential involvement of kainate receptor subunits in LTP of spinal DH neurons.

## 4. Voltage-Gated Ca^**2+**^ Channels That Contribute to LTP in Spinal DH and to Pain

Although receptors for L-glutamate, most commonly the NMDA receptor subtype, mediate induction and expression of LTP at many synapses in the brain, some forms of LTP at hippocampal CA1 synapses, such as late-phase LTP (L-LTP), require activation of L-type VGCCs. Other VGCCs are also involved in diverse ways in LTP, as discussed below.

VGCCs consist of a pore-forming transmembrane *α*_1_ subunit and the auxiliary *β* subunit and *α*_2_ *δ* subunit [185]. Based on sequence homology, the ten different *α*_1_ subunits of VGCCs are grouped into three subfamilies: two high-voltage activated subfamilies, Ca_V_ 1-2, and one low-voltage activated family, Ca_V_3 [186]. The Ca_V_1 subfamily carries L-type Ca^2+^ current, and the family members are Ca_V_1.1 (*α*_1S_), Ca_V_1.2 (*α*_1C_), Ca_V_1.3 (*α*_1D_), and Ca_V_1.4 (*α*_1F_). The Ca_V_2 subfamily includes Ca_V_2.1 (*α*_1A_), Ca_V_2.2 (*α*_1B_), and Ca_V_2.3 (*α*_1E_) which correspond to P/Q-type, N-type, and R-type Ca^2+^ currents, respectively. The Ca_V_3 subfamily carries T-type Ca^2+^ currents, and the family members are Ca_V_3.1 (*α*_1G_), Ca_V_3.2 (*α*_1H_), and Ca_V_3.3 (*α*_1I_) [186]. The channel's auxiliary subunits are also organized into subfamilies, and these specifically affect membrane trafficking and expression of channels, voltage-dependence of channel opening, inactivation kinetics, and sensitivities to inhibitors, thus greatly expanding the number of different subtypes of VGCCs [185, 187]. In this section, we will discuss the contribution of each major type of VGCC to LTP in the spinal DH to various forms of pain in normal and pathological states.

### 4.1. L-Type VGCCs

#### 4.1.1. Contribution to LTP

L-type VGCCs are widely expressed in the CNS [188], including CA1 of the hippocampus, the preeminent region for investigations of LTP. The dendritic localization of L-type VGCCs in the CA1 area [189] implies that their activation contributes to Ca^2+^ signals in dendritic spines, an important step for the induction of LTP [190]. Although induction of canonical LTP in CA1 relies upon Ca^2+^ flux through NMDA receptors on dendritic spines and subsequent activation of Ca^2+^-dependent second messengers [70], several other forms of LTP in fact require activation of L-type VGCCs, and not NMDA receptors. In the hippocampal CA1 area, for example, HFS at 200 Hz [191], (higher frequency than the 100 Hz tetani typically employed for induction of NMDA receptor-dependent LTP) generates LTP that is insensitive to the NMDA receptor antagonist D-AP5 but is blocked by the L-type VGCC antagonist, nifedipine. While NMDA receptor-dependent LTP is inhibited by antagonists of serine-threonine kinases, the 200 Hz-induced, L-type VGCC-dependent LTP is blocked by antagonists of tyrosine kinases [192]. In addition, prolonged theta burst stimulation (TBS) in the CA1 area induces a form of LTP that is dependent upon L-type VGCCs [193]. L-type VGCC-dependent LTP can also be produced by application of the potassium channel blocker, tetraethylammonium (TEA) [194, 195]. Interestingly, the mechanism of induction of this type of LTP partially overlaps that of NMDA receptor-dependent LTP, particularly in regard to the timing and intensity of postsynaptic Ca^2+^ signals [195]. Extracellular matrix molecules, such as hyaluronic acid and tenascin-R, are important in the development of L-type VGCC-dependent LTP induced by either TBS [196] or TEA [197]. As for Schaffer collateral-CA1 pyramidal neuron synapses, L-channel-dependent LTP has been described at mossy fiber synapses onto CA3 pyramidal neurons [198] and for thalamic inputs to amygdala [199]. Induction of NMDA receptor-independent, L-channel-dependent LTP is distinctive in its reliance upon such electrical phenomena as dendritic Ca^2+^ spikes [200–202].

In spinal DH, although L-type VGCCs are known to be expressed in soma, dendrites, and axon terminals of neurons [203, 204], it appears that L-channels do not induce LTP during 100 Hz repetitive stimulation [98]. L-type VGCCs contribute instead to an alternative form of LTP in spinal lamina I neurons, one that is induced by postsynaptic depolarization without presynaptic stimulation (“non-Hebbian” LTP) [205]. Gabapentin, which binds to the *α*_2_ *δ* subunit of VGCCs and is used to relieve neuropathic pain, does not affect C fiber-mediated basal transmission or LTP induction but does reduce C fiber-mediated transmission during the maintenance phase of LTP [206]. Thus, postsynaptic Ca^2+^ influx through L-type VGCCs may be critical to induce or express LTP of excitatory synaptic transmission in certain normal and/or pathological states. More extensive investigation of distinct types of LTP induced under normal or neuropathological conditions is clearly needed to better understand the contribution of L-type VGCCs to synaptic plasticity and neuropathic pain of the spinal DH.

#### 4.1.2. Contribution to Pain

Implication of L-type VGCCs in acute and chronic pain has been controversial. Some reports show that spinal administration of L-type VGCC blockers decreases pain sensitivity to acute innocuous or noxious stimuli [207, 208], but other work has found no effect of these blockers in the hot plate test [209] or in other tests using acute mechanical and thermal stimuli [210, 211]. Furthermore, in a chronic pain model of peripheral nerve injury, intrathecal administration of a high dose of the L-type VGCC blocker, diltiazem, has no effect on paw withdrawal in response to mechanical stimulation [212].

 Recently, however, it has been found that prolonged intrathecal administration of the L-channel blocker nicardipine elevates mechanical threshold in a neuropathic pain model [213], indicating the involvement of L-type VGCCs in mechanical allodynia caused by peripheral nerve injury. Along the same lines, reduced expression of L-type VGCCs in spinal DH by antisense [213] or microRNA [214] technologies suppresses the hypersensitivity of DH neurons following peripheral nerve injury. Taken together, these findings indicate that L-type VGCCs can contribute to some components of acute or chronic pain behaviors produced by tissue damage, likely reflecting the contribution of L-type VGCCs to certain forms of LTP in the spinal DH.

### 4.2. P/Q-Type VGCCs

#### 4.2.1. Contribution to LTP

P/Q-type VGCCs are expressed in a subpopulation of DRG neurons [203, 215, 216] that does not respond to capsaicin and rarely expresses substance P, a marker for small high-threshold primary afferent terminals [203]; P/Q channels thus play only a small role in the control of glutamate release from small diameter, peptidergic nociceptive primary afferent fibers. In addition, it has been suggested that P/Q-type VGCCs are expressed in fewer numbers of primary afferents than are N-type VGCCs [203, 217, 218]. In the spinal DH, P/Q-type VGCCs are expressed in the laminae II–VI [203] and are preferentially involved in inhibitory neurotransmission [219, 220], indicating a limited contribution of P/Q-type VGCCs to excitatory synaptic transmission in the spinal DH.

 Blockers of P/Q-type VGCCs strongly suppress induction of LTP for C fiber-evoked field potentials [90], suggesting that induction of this form of spinal DH LTP may rely in part upon P/Q-type VGCCs [221]. Similarly, in visual cortical neurons, P/Q-type VGCCs have also been proposed to contribute to the induction of LTP at the inhibitory synapses [222].

#### 4.2.2. Contribution to Pain

In accord with the minimal contribution of P/Q-type VGCCs to glutamate release from small-diameter, high-threshold primary afferents [203], intrathecal administration of the selective P/Q channel blocker, *ω*-agatoxin IVA, has little or no effect on C- or A*δ* fiber-mediated nociceptive transmission [223] or in tests of mechanical and thermal thresholds in neuropathic pain models [212, 224]. However, development of hyperalgesia or pathological pain is prevented by intrathecal pretreatment with blockers of P/Q-type VGCCs [209–211], as well as in animals with either a genetic deficiency [225] or spontaneous mutation [226] in P/Q-type VGCCs. These observations correlate with studies of spinal LTP that indicate a critical role for P/Q-type VGCCs in the induction of LTP of C fiber-evoked field potentials [90]. Therefore, as for L-type VGCCs, P/Q-type VGCCs may also be involved in the development or regulation of certain forms of chronic pain.

### 4.3. N-Type VGCCs

#### 4.3.1. Contribution to LTP

N-type VGCCs are expressed in dorsal root ganglia, as well as in primary afferent nerve terminals in the superficial area (laminae I-II) of DH [203, 217]. In accord with these findings, glutamatergic transmission between DRG and spinal DH neurons is blocked by *ω*-conotoxin GVIA, a selective blocker of N-type VGCCs [227]. Many of the presynaptic nerve terminals with N-type VGCC immunoreactivity also contain substance P, suggesting that N-type channels also support the release of substance P and CGRP from peptidergic, high-threshold C fibers in the spinal DH [228, 229].

As for other VGCCs, the involvement of N-type VGCCs in synaptic plasticity, particularly LTP, appears to be specific to the synaptic pathway and induction protocol. An early study showed that when a 100 Hz induction protocol is used, N-type VGCCs are not involved in LTP of the hippocampal mossy fiber-mediated CA3 pathway [201]. However, at hippocampal CA3-to-CA1 Schaffer collateral synapses, when TBS or 200 Hz HFS is used to induce LTP, N-channel-mediated component of excitatory transmission can be identified after induction of LTP [230]. For perforant path synapses onto CA1 neurons, induction of LTP by 200 Hz HFS relies upon an increased contribution from presynaptic N-type VGCCs [231]. In the spinal DH, the N-channel blocker *ω*-conotoxin GVIA does not prevent induction of LTP of C fiber-field potentials, but this N channel antagonist inhibits synaptic transmission once LTP has been induced [90]. These results indicate that presynaptic N-type channels contribute to the maintenance phase of LTP in the spinal DH [231].

#### 4.3.2. Contribution to Pain

Implication of N-type VGCCs in acute and chronic pain states correlates with the fact that N-type channels are predominantly expressed in DRG neurons, particularly small-diameter peptidergic DRG neurons and the fact that N channels may control release of neurotransmitters such as glutamate and substance P onto spinal DH neurons [228, 229]. Antagonists of N-type channels block the release of glutamate, substance P, and CGRP in the spinal DH [229, 232, 233], and in various acute, inflammatory, and neuropathic pain models, produce antinociceptive and analgesic effects [234, 235]. In addition, genetic ablation of Ca_V_2.2 (*α*_1B_), the pore-forming subunit of N-type channels, significantly reduces mechanical allodynia and thermal hyperalgesia in a neuropathic pain model with spinal nerve ligation [236]. Moreover, the antinociceptive effects of N-channel antagonists are enhanced in chronic pain states [237, 238], as N-type channels are upregulated after peripheral nerve injury [239]. Interestingly, these findings correlate with the observation that the block of N-channels inhibits synaptic transmission once LTP has been established [90].

 Even though neuropathic pain can be reduced by blockers of N-type channels, and blockers such as *ω*-conotoxin MVIIA (SNX-111, ziconotide, or Prialt) have received approval by the FDA for the treatment of chronic pain; N-channel blockers are of limited use owing to side effects attributable to the fact that almost all of the presynaptic terminals in the brain express the N-type VGCCs. Thus, N-channel antagonists must be administered intrathecally, an invasive method used when other pain management options have failed. It may therefore prove useful to develop N-channel blockers that are either specific to particular Ca_V_1.2 *α*_1B_ splice variants [240, 241] or that modulate N-type channel in selective neuronal subtypes [242].

### 4.4. T-Type VGCCs

#### 4.4.1. Contribution to LTP

Although the lack of a specific antagonist prevents clean isolation of the contribution of T-type VGCCs to LTP, there is evidence indicating that T-type VGCCs are involved in the induction and/or expression of LTP in hippocampal CA1 [243] neurons and dentate gyrus [244]. The late phase of LTP in the CA1 area, which can be induced by 100 Hz HFS and is dependent on NMDA receptors, is not maintained (<120 min) in Ca_V_3.2 T-type VGCC knockout mice [243]. LTP induction in dentate gyrus is sensitive to Ni^2+^, a blocker of T-type as well as R-type VGCCs, and is dependent on the induction protocol: the Ni^2+^-sensitive component of LTP can be induced by pairing of 1 Hz presynaptic stimulation with postsynaptic depolarization, but not by 100 Hz HFS [244]. T-type VGCCs may also be involved in LTP that is induced by TBS [245] or TEA [246] in the CA1 area, although L-channels have alternatively been reported to mediate TEA-induced LTP [195]. T-type VGCCs in CA1 are implicated in the enhancement of LTP, rather than its induction or expression, with the mechanism involving muscarinic acetylcholine receptors and phospholipase C-mediated K^+^ channel inhibition [247].

T-type VGCCs are expressed in the superficial DH of the spinal cord, as well as in medium- and small-diameter DRG neurons [248], raising the possibility of both pre- and postsynaptic contributions to synaptic plasticity at primary afferent-DH neuron synapses. T-type Ca^2+^ currents have been reported in spinal DH neurons [223, 249–253]. In addition, the contribution of T-type Ca^2+^ channels to LTP of C fiber-initiated EPSCs in lamina I neurons that project to the PB area has been demonstrated [99], suggesting that T channels may play a significant role in amplification of pain signals via their contribution to spinal LTP. On the other hand, LFS-induced LTP of C fiber-EPSCs in PAG-projecting lamina I neurons [87] likely involves T-type VGCCs [254]. These results underscore the idea, once again, that spinal LTP is cell type-specific.

#### 4.4.2. Contribution to Pain

The involvement of T-type VGCCs in pain is likely specific to T-channel subtypes. Nociceptive responses induced by nerve injury are decreased after knock down of Ca_V_3.2, but not of Ca_V_3.1 or Ca_V_3.3 [255], presumably reflecting the abundance of Ca_V_3.2 in DRG neurons [255–257] and indicating that the T-channel subtype involved in spinal LTP may be Ca_V_3.2. In support of this interpretation, genetic knockout of Ca_V_3.2 attenuates behavioral responses to noxious stimuli such as formalin [258]. In contrast, knockout of Ca_V_3.1 causes hypersensitivity to noxious visceral stimuli, but this involves a supraspinal mechanism [259]. Therefore, although some nonselective T-type blockers such as ethosuximide or mibefradil can reverse both tactile hypersensitivity and thermal hyperalgesia in various pain models [260, 261], development of subtype-specific antagonists of T-type channels is desirable. In this regard, downregulation of T-type channel activity and of hyperalgesia by oxidizing agents [262, 263] or by lowering levels of the endogenous gasotransmitter hydrogen sulfide [264, 265] may prove useful as leads in developing novel subtype-selective T-channel drugs for the treatment of inflammatory or neuropathic pain.

### 4.5. R-Type VGCCs

#### 4.5.1. Contribution to LTP

The involvement of R-type VGCCs in LTP can be difficult to isolate, in part because a commonly-used R-type channel blocker Ni^2+^ is also an effective blocker of T-type channels. However, there is strong evidence that R-type channels support a presynaptic form of LTP found at parallel fiber synapses onto Purkinje cells in the cerebellum [266]. Because cerebellar granule cells (which give rise to parallel fibers) do not express T-type channels, in this system, the effects of Ni^2+^ block of Ca^2+^ current can be entirely attributed to antagonism of R-type channels. In comparison to N- and P/Q-channel-mediated Ca^2+^ influx at parallel fiber terminals, R-type channels contribute only modestly to bulk changes in intracellular Ca^2+^, suggesting that R-channel Ca^2+^ microdomains in presynaptic terminals are important for the induction of parallel fiber LTP [266]. 

On the postsynaptic side, R-type VGCCs in CA1 pyramidal neurons contribute to Ca^2+^ influx evoked by TBS-triggered, back-propagating dendritic action potentials [267, 268]. In turn, R-channel Ca^2+^ influx in distal dendrites of CA1 pyramidal neurons helps generate plateau potentials that are critical for perforant path LTP [269].

Although R-type VGCCs are expressed in a subpopulation of DRG neurons [270], it is unclear whether the primary afferent terminals or spinal DH neurons bear R-type channels. In addition, whether R-type channels are involved in synaptic plasticity in the spinal DH remains to be determined.

#### 4.5.2. Contribution to Pain

There is evidence that R-type VGCCs are involved in the transmission and processing of inflammatory and neuropathic pain information. SNX-482, an inhibitor of R-type VGCCs (and less potently of L-type channels) [271], decreases nociceptive responses during the second phase of the formalin test [217] and inhibits neuropathic pain behavior [272]. In addition, studies using Ca_V_2.3 knockout mice suggest a contribution of R-type VGCCs to pain transmission [217, 273].

## 5. Concluding Remarks

Many studies have attempted to elucidate rules and signaling mechanisms for synaptic plasticity, particularly LTP, in the spinal DH. Together, these studies show that LTP in the spinal DH shares many features with LTP in the hippocampus and with “central sensitization” in the spinal DH during hyperalgesia [274]. In this review, we have considered how inter-relationships between synaptic circuitry in the spinal DH, ionotropic glutamate receptors, voltage-gated Ca^2+^ channels, and induction/expression of LTP in the spinal DH are together involved in pain hypersensitivity.

Up to the present time, the complexity of synaptic circuitry in the spinal DH has hampered the understanding of LTP mechanisms in spinal DH. Generally, thorough classification of postsynaptic neurons and presynaptic fibers remains to be worked out. Although presynaptic fiber type can be identified during electrophysiological recording based upon conduction velocity and stimulus intensity, selective stimulation of a single class of primary afferent fiber remains challenging, because the range of stimulus intensities that activate C fibers overlaps the range of intensities that activates A fibers. Postsynaptically, in the spinal DH, although multiple morphological and electrophysiological criteria are available to distinguish neuronal subtypes, studying synaptic plasticity in a single type of spinal postsynaptic neuron has yet to be achieved, owing to inhomogeneities in physiological behavior, neurotransmitters, and cellular markers even within a group of neurons that carries out a similar function, such as the lamina I projection neurons [7]. 

In the future, progress in this field will likely rely upon studies that make use of powerful new experimental approaches, such as combining transgenic means to identify postsynaptic neurons [29, 32] and presynaptic fibers [25] with optogenetic tools to selectively activate specific fiber types [275]. This kind of approach will make it possible to study LTP at synapses between specific types of primary afferent fibers and spinal DH neurons or between specific spinal DH neurons, thereby facilitating the correlation between mechanisms of LTP and nociception in the spinal DH.
